# Maximal isokinetic elbow and knee flexor–extensor strength measures in combat sports athletes: the role of movement velocity and limb side

**DOI:** 10.1186/s13102-022-00432-2

**Published:** 2022-03-16

**Authors:** Said El-Ashker, Helmi Chaabene, Olaf Prieske

**Affiliations:** 1 Self-Development Department, Deanship of Preparatory Year, Imam Abdulrahman Bin Faisal University, Dammam, 31441 Saudi Arabia; 2 Department of Sports and Health Sciences, Faculty of Human Sciences, University of Potsdam, 14469 Potsdam, Germany; 3 High Institute of Sports and Physical Education, Kef, University of Jendouba, 8189 Jendouba, Tunisia; 4 Division of Exercise and Movement, University of Applied Sciences for Sports and Management Potsdam, Potsdam, Germany

**Keywords:** Dynamometry, Martial arts, Hamstring-quadriceps ratio, Eccentric muscle action, Injury

## Abstract

**Background:**

Maximal isokinetic strength ratios of joint flexors and extensors are important parameters to indicate the level of muscular balance at the joint. Further, in combat sports athletes, upper and lower limb muscle strength is affected by the type of sport. Thus, this study aimed to examine the differences in maximal isokinetic strength of the flexors and extensors and the corresponding flexor–extensor strength ratios of the elbows and knees in combat sports athletes.

**Method:**

Forty male participants (age = 22.3 ± 2.5 years) from four different combat sports (amateur boxing, taekwondo, karate, and judo; n = 10 per sport) were tested for eccentric peak torque of the elbow/knee flexors (EF/KF) and concentric peak torque of the elbow/knee extensors (EE/KE) at three different angular velocities (60, 120, and 180°/s) on the dominant and non-dominant side using an isokinetic device.

**Results:**

Analyses revealed significant, large-sized group × velocity × limb interactions for EF, EE, and EF–EE ratio, KF, KE, and KF–KE ratio (*p* ≤ 0.03; 0.91 ≤ d ≤ 1.75). Post-hoc analyses indicated that amateur boxers displayed the largest EE strength values on the non-dominant side at ≤ 120°/s and the dominant side at ≥ 120°/s (*p* < 0.03; 1.21 ≤ d ≤ 1.59). The largest EF–EE strength ratios were observed on amateur boxers’ and judokas’ non-dominant side at ≥ 120°/s (*p* < 0.04; 1.36 ≤ d ≤ 2.44). Further, we found lower KF–KE strength measures in karate (*p* < 0.04; 1.12 ≤ d ≤ 6.22) and judo athletes (p ≤ 0.03; 1.60 ≤ d ≤ 5.31) particularly on the non-dominant side.

**Conclusions:**

The present findings indicated combat sport-specific differences in maximal isokinetic strength measures of EF, EE, KF, and KE particularly in favor of amateur boxers on the non-dominant side.

*Trial registration*: This study does not report results related to health care interventions using human participants and therefore it was not prospectively registered.

## Background

Muscle strength is one of the major physical fitness components for successful performance and health (e.g., injury prevention) in many sports [1]. In combat sports, well-developed upper and/or lower limb muscle strength is key to effectively manage the high physical and technical/tactical demands of the competition in amateur boxing [2], taekwondo [3], karate [4], and judo [5]. For instance, isokinetic lower limb muscle strength measures were significantly larger in regional karate athletes compared with active, non-karate controls [6]. Additionally, elite judo athletes exhibited higher strength/power performance (e.g., maximal force and power during sport-specific pulling tests) compared with sub-elite athletes [7].

Further, in terms of health, higher upper and lower limbs muscle strength may also have injury-preventive effects in combat sports athletes [8]. Of note, earlier studies indicated that most injuries in combat sports occur at upper and lower limb extremities [8–11]. For instance, a systematic review of Pocecco et al. [8] found that injuries of the knee (e.g., rupture of the anterior cruciate ligament [ACL]) were amongst the most frequent injuries in judokas (i.e., incidence rate up to 28%). Interestingly, lower knee flexor (KF) relative to knee extensor (KE) strength has been discussed as a potential mechanism for increased lower limb injuries such as ACL tears [12, 13]. In fact, KF and KE strength play a crucial role in affecting anterior tibial translation and ACL strain [14, 15]. Furthermore, alongside the needed high levels of (lower limb) muscle strength, the good balance between agonists’ and antagonists’ strength levels (e.g., quadriceps vs. hamstrings) is an additional key factor that contributes to mitigating the risk of injuries (e.g., ACL tear) in combat sports athletes [8, 16].

Generally, the specific needs of the respective sport dictate the level of physical fitness (e.g., muscle strength) that is required [4]. In this regard, amateur boxers performed mainly punches [2]. The capability of boxers to deliver successful punches depends on upper/lower limb muscle power and strength [2]. In contrast, taekwondo athletes mainly use kicks during the competition [17], whereas karate athletes use both punches and kicks with a greater emphasis on the former [18, 19]. Lastly, judo athletes used upper more than lower limb muscles predominantly during pulling movements when compared with the other combat sports [5]. Consequently, the level of upper and lower limb muscle strength and the respective strength ratios may differ between striking (e.g., amateur boxing, karate, and taekwondo) and grappling combat sports (e.g., judo). Of note, previous studies showed inconsistent findings in maximal upper and lower limb muscle strength between boxers, judoka, and taekwondo athletes [20–22]. While Busko [21] demonstrated similar maximal isometric strength of the left and right elbow, knee and hip flexors and extensors in male boxers, judoka, and taekwondo athletes, Szafranski and Boguszewski [22] revealed significantly higher maximal isokinetic muscle strength of the dominant and non-dominant KE at 240°/s in kickboxers compared with taekwondo athletes. It seems reasonable to assume that dynamic (e.g., isokinetic) muscle strength testing using higher (angular) velocities may adhere more properly to the specific characters of the combat sports. Further, the effect of limb side on group differences in (maximal isokinetic) muscle strength is yet to be examined.

Thus, this study aimed at examining maximal isokinetic upper (i.e., elbow flexors [EF]/extensors [EE]) and lower limb muscle (i.e., KF/KE) strength and the corresponding maximal isokinetic flexor–extensor strength ratios of the dominant and non-dominant side at different angular velocities (i.e., 60°/s, 120°/s, and 180°/s) in athletes from various combat sports (i.e., amateur boxing, taekwondo, karate, and judo). With reference to the relevant literature [2, 5, 17–19, 22], we hypothesized (1) sport-specific differences in maximal isokinetic upper and lower limb muscle strength in athletes from different combat sport (e.g., larger maximal EF/EE strength in boxers and judokas but larger maximal KF/KE strength in karate and taekwondo athletes) and (2) that these differences become more apparent at higher angular velocities.

## Methods

### Participants

Forty male combat sports athletes (amateur boxing [n = 10], karate [n = 10], taekwondo [n = 10], and judo [n = 10]) with 4.5 ± 1.5 years of training experience, volunteered to participate in this study. With reference to the study of Szafranksi and Boguszewski [22], an a priori power analysis [23] with an assumed Type I error of 0.05 and a Type II error rate of 0.20 (80% statistical power) was calculated for maximal isokinetic KE strength and revealed that 40 participants would be sufficient to observe at least small-sized interaction effects between factors group, angular velocity, and/or limb side. Detailed characteristics of the recruited combat sports athletes are displayed in Table 1. Participants performed 3 ± 1 training sessions per week with each session lasting 100 ± 25 min. The present study was approved by the local ethics committee under the following number “IRB-2018-19-112”. Furthermore, all participants were fully informed about the aim and potential risks of the study and agreed to participate by signing a written informed consent.

**Table 1:** Characteristics of the participating combat sports athletes

	Boxing	Taekwondo	Karate	Judo	*p* value
Age (years)	22.5 ± 2.6	22.7 ± 2.45	21.9 ± 2.02	22.2 ± 2.14	0.835
Body height (cm)	176.0 ± 4.8	174.6 ± 3.9	173.7 ± 3.9	173.6 ± 7.6	0.370
Body mass (kg)	76.6 ± 5.7	76.9 ± 4.70	72.3 ± 6.16	76.2 ± 7.6	0.276
Body mass index (kg/m^2^)	24.7 ± 1.25	25.2 ± 1.19	23.9 ± 1.18	25.2 ± 0.91	0.071
Training experience (years)	4.5 ± 0.84	4.4 ± 0.85	4.5 ± 1.08	4.4 ± 0.83	0.993

### Procedure

To examine maximal upper and lower limb muscle strength in athletes from different combat sports, a cross-sectional study design was used. Maximal strength measurements were conducted using an isokinetic dynamometer (Biodex System Pro, Biodex Medical Systems, USA). In this regard, all participants were asked to attend two separate testing sessions 2–3 days apart. The first session was used for familiarization and the second was used for the assessment of maximal isokinetic upper and lower limb muscle strength. During familiarization, the anthropometric characteristics (e.g., body mass and height) of participants were determined. Additionally, two to three concentric and eccentric submaximal testing trials on the isokinetic device were performed during the familiarization session with upper and lower limbs of the dominant and non-dominant side at 120°/s to get accustomed to the testing protocol [24, 25]. Before the testing and measurement trials (i.e., second session), participants performed 10 min warm-up using a cycle ergometer (Monark Exercise, Vansbro, Sweden) at a self-selected intermediate intensity workload, with a rating of perceived exertion (RPE) of 5–6 points on a 0–10 RPE scale, followed by 5 min of dynamic and static quadriceps and hamstring stretching [25]. For the upper limb muscles, 3–5 min of unloaded upper body cycling was performed using an upper-body cycle ergometer (Saratoga Cycle, Rand-Scot, CO, USA) [26]. The same warm-up protocol was used before the assessment of the participants’ maximal isokinetic upper and lower limb muscle strength. The order of upper and lower limb muscle testing was randomized.

### Assessment of maximal isokinetic upper limb muscle strength

For the assessment of maximal isokinetic EF and EE strength, participants were seated in a rigid chair of the isokinetic dynamometer with shoulders flexed at 40°. Straps attached to the isokinetic system firmly fixed the upper body and the hip. The elbow joint was aligned to the dynamometer’s axis [27] with the forearm in a neutral position [28]. The range of motion was set at 20° to 130° with 0° corresponding to full extension [29]. Participants performed three repetitions of maximal concentric EE and eccentric EF actions in a standardized order of angular velocity (i.e., 60°/s, 120°/s, and 180°/s) with 1 min of rest between velocities. Assessments started with the participants’ dominant side followed by the non-dominant side. Hand dominance was determined according to the lateral preference inventory [30]. The torque signal was corrected for gravity. The highest torque values during the three repetitions were used for further analysis. Maximal isokinetic strength was defined as concentric EE peak torque (PT) and eccentric EF PT. Additionally, the functional EF–EE strength ratio was calculated as eccentric EF PT divided by concentric EE PT [31]. Pilot testing indicated good test–retest reliability for maximal isokinetic upper limb muscle strength measures with intraclass correlation coefficients (ICC) ranging from 0.83 to 0.91.

### Assessment of maximal isokinetic lower limb muscle strength

For the assessment of maximal isokinetic KF and KE strength, participants were stabilized on the dynamometer in a supine position for the concentric knee extension actions and in a prone position for the eccentric knee flexion actions (hip flexion: 10°–20°) [14]. The mechanical axis of the dynamometer’s lever arm was adjusted to the lateral epicondyle of the knee joint. The shank was attached to the dynamometer approximately 3-cm superior to the medial malleolus with the foot in a relaxed position. Adjustable straps across the pelvis, thigh proximal to the knee and foot were used for fixation. The knee joint range of motion was set at 0°–90° (0° represents full knee extension). Similar to upper limbs, measurements were performed with three repetitions of maximal concentric KE and eccentric KF actions in a standardized order of angular velocity (i.e., of 60°/s, 120°/s, and 180°/s) with 1 min of rest in-between. Assessments started with the participants’ dominant side followed by the non-dominant side. Leg dominance was determined according to Coren [30]. The torque signal was corrected for gravity. The highest torque values during the three repetitions were used for further analysis. Maximal isokinetic strength was defined as concentric KE PT and eccentric KF PT. The functional KF–KE strength ratio (i.e., hamstring-quadriceps ratio) was calculated as eccentric KF PT divided by concentric KE PT [32]. Test reliability for maximal isokinetic lower limb muscle strength measures was good (0.85 ≤ ICC ≤ 0.94).

### Statistical analyses

Data are presented as means ± standard deviation (SD). After normal distribution was verified using the Shapiro–Wilk test, a 4 (group: karate, judo, amateur boxing, taekwondo) × 3 (velocity: 60°/s, 120°/s, 180°/s) × 2 (side: dominant, non-dominant) analysis of variance (ANOVA) with repeated measures on velocity and side was used to examine the effects of combat sports group, angular velocity, and limb side on maximal isokinetic strength of the flexors and extensors as well as isokinetic flexor–extensor strength ratios of the knee and elbow joints. To examine the homogeneity of variance between groups, Levene’s test was used. Additionally, sphericity was tested using the Mauchly test. The Greenhouse–Geisser correction was applied for further analysis if sphericity was violated. In the case of significant interaction effects, post-hoc tests with Bonferroni-adjusted α values were conducted to identify paired comparisons that reached statistical significance. The significance level was set at *p* < 0.05. In terms of participants’ characteristics, a one-way (group: karate, judo, amateur boxing, taekwondo) ANOVA was performed. Additionally, effect sizes were determined by converting partial eta^2^ to Cohen’s *d* [33]. According to Cohen, effect sizes are classified as small (0.0 ≤ *d* < 0.5), medium (0.5 ≤ *d* < 0.8), and large (*d* ≥ 0.8). Reliability was determined by calculating the ICC. We considered an ICC < 0.50 as poor, 0.50 ≤ ICC < 0.75 as moderate, 0.75 ≤ ICC < 0.90 as good, and ICC > 0.90 as excellent [34]. All statistical analyses were performed using SPSS (v.25.0; IBM, Armonk, NY, USA).

## Results

### Maximal isokinetic upper limb muscle strength

Means and standard deviations of maximal isokinetic upper limb muscle strength measures are presented in Table 2. Our statistical analysis revealed significant main effects of velocity and side on eccentric EF PT, concentric EE PT, and EF–EE strength ratio (*p* ≤ 0.001, 1.57 ≤ *d* ≤ 8.13). Significant main effects of group were found for eccentric EF PT and concentric EE PT (*p* ≤ 0.010, 1.20 ≤ *d* ≤ 2.62) (Table 3).

**Table 2:** Maximal isokinetic strength measures of elbow flexors and extensors of the dominant (D) and non-dominant (ND) side at 3 angular velocities (60, 120, and 180°/s) in combat sport athletes

	Karate		Judo		Boxing		Taekwondo	
	D	ND	D	ND	D	ND	D	ND
*Maximal eccentric elbow flexor peak torque (Nm)*								
60°/s	51.8 ± 3.3	49.2 ± 3.2	57.3 ± 2.3	53.9 ± 2.7	61.7 ± 5.4	59.0 ± 5.5	55.0 ± 2.8	51.3 ± 3.8
120°/s	43.0 ± 3.3	37.3 ± 3.1	48.7 ± 2.7	45.5 ± 2.2	51.9 ± 4.9	48.7 ± 5.4	47.8 ± 3.1	43.0 ± 2.9
180°/s	40.1 ± 3.3	36.9 ± 2.6	44.8 ± 3.0	42.1 ± 2.1	46.8 ± 2.0	44.7 ± 2.5	41.9 ± 1.9	39.4 ± 2.0
*Maximal concentric elbow extensor peak torque (Nm)*								
60°/s	40.3 ± 4.6	34.6 ± 3.7	42.9 ± 3.8	38.1 ± 3.9	45.5 ± 6.5	42.2 ± 7.6	41.8 ± 4.1	36.5 ± 3.9
120°/s	33.5 ± 3.0	30.8 ± 2.8	37.4 ± 2.5	34.2 ± 2.6	39.8 ± 4.7	35.0 ± 4.0	34.2 ± 2.4	32.2 ± 2.3
180°/s	33.4 ± 4.3	31.8 ± 4.1	35.6 ± 3.4	33.4 ± 2.6	38.0 ± 3.2	35.6 ± 2.2	36.4 ± 2.9	34.5 ± 2.9
*Elbow flexor–extensor strength ratio*								
60°/s	1.30 ± 0.14	1.43 ± 0.17	1.34 ± 0.08	1.42 ± 0.09	1.37 ± 0.10	1.42 ± 0.16	1.32 ± 0.10	1.41 ± 0.12
120°/s	1.29 ± 0.07	1.21 ± 0.09	1.30 ± 0.03	1.33 ± 0.06	1.31 ± 0.09	1.39 ± 0.05	1.40 ± 0.04	1.34 ± 0.10
180°/s	1.21 ± 0.09	1.17 ± 0.09	1.27 ± 0.15	1.27 ± 0.10	1.24 ± 0.09	1.26 ± 0.08	1.16 ± 0.06	1.15 ± 0.07

**Table 3:** Effects of group, velocity, and limb on maximal isokinetic strength measures of elbow flexors and extensors in combat sport athletes

	(G)roup		(V)elocity		(L)imb		G × V		G × L		V × L		G × V × L	
	p	d	p	d	p	d	p	d	p	d	p	d	p	d
Maximal eccentric elbow flexor peak torque	< 0.001	2.62	< 0.001	6.10	< 0.001	7.23	0.161	0.73	0.041	1.01	< 0.001	1.13	0.030	0.91
Maximal concentric elbow extensor peak torque	0.010	1.20	< 0.001	3.01	< 0.001	8.13	0.135	0.75	0.717	0.39	< 0.001	1.93	< 0.001	1.37
Elbow flexor–extensor strength ratio	0.159	0.78	< 0.001	2.20	< 0.001	1.57	0.025	0.93	0.016	1.14	< 0.001	1.95	< 0.001	1.64

*d* = effect size (Cohen’s d)

**Fig. 1:**
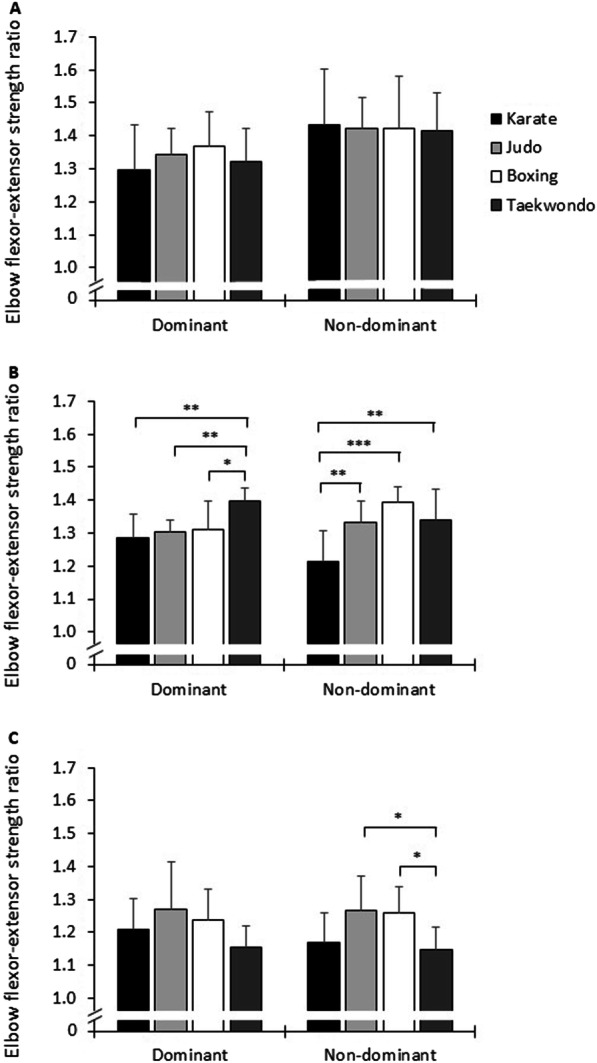
Elbow flexor–extensor strength ratios at **a** 60°/s, **b** 120°/s, and **c** 180°/s on the dominant and non-dominant sides in combat sports athletes

Additionally, we observed significant interaction effects of group, velocity, and side on eccentric EF PT, concentric EE PT, and EF–EE strength ratio (*p* ≤ 0.030, 0.91 ≤ *d* ≤ 1.64). Post-hoc analysis showed lower eccentric EF PT values in karate and taekwondo athletes compared with judoka and boxers predominantly at 120°/s and 180°/s on the non-dominant side (*p* ≤ 0.010, 1.48 ≤ *d* ≤ 3.04). In terms of EE, post-hoc analysis showed higher concentric PT in boxers compared with karate athletes at 60°/s on the non-dominant side (*p* = 0.010, *d* = 1.28), at 180°/s on the dominant side (*p* = 0.034, *d* = 1.21), and at 120°/s (*p* ≤ 0.020, 1.22 ≤ *d* ≤ 1.59), irrespective of limb side. Moreover, while post-hoc analysis showed higher EF–EE strength ratios for taekwondo athletes compared with judokas, amateur boxers, and karate athletes at 120°/s on the dominant side (*p* ≤ 0.015, 1.33 ≤ *d* ≤ 2.64), ratios on the non-dominant side were higher in judokas and amateur boxers compared with karate athletes (120°/s) and taekwondo athletes (180°/s) (*p* ≤ 0.039, 1.36 ≤ *d* ≤ 2.44) (Fig. 1).

### Maximal isokinetic lower limb muscle strength

Means and standard deviations of maximal isokinetic lower limb muscle strength measures are presented in Table 4. Our statistical analysis revealed significant main effects of velocity and side on eccentric KF PT, concentric KE PT, and KF–KE strength ratio (*p* ≤ 0.019, 0.82 ≤ *d* ≤ 20.99). Significant main effects of group were found for concentric KE PT and KF–KE strength ratio (*p* ≤ 0.010, 1.20 ≤ *d* ≤ 1.39) (Table 5).

**Table 4:** Maximal isokinetic strength measures of knee flexors and extensors of the dominant (D) and non-dominant (ND) side at 3 angular velocities (60, 120, and 180°/s) in combat sport athletes

	Karate		Judo		Boxing		Taekwondo	
	D	ND	D	ND	D	ND	D	ND
*Maximal eccentric knee flexor peak torque (Nm)*								
60°/s	153.8 ± 13.8	143.1 ± 5.0	159.0 ± 14.0	145.5 ± 7.4	165.9 ± 22.2	161.0 ± 13.4	162.0 ± 12.5	148.0 ± 5.0
120°/s	124.3 ± 12.9	119.4 ± 11.1	127.4 ± 10.6	120.9 ± 7.7	130.8 ± 15.8	122.8 ± 9.8	128.9 ± 8.6	107.7 ± 6.1
180°/s	97.2 ± 6.4	90.3 ± 6.8	102.6 ± 5.1	91.4 ± 3.3	105.5 ± 10.8	84.1 ± 9.7	103.0 ± 5.4	88.3 ± 4.4
*Maximal concentric knee extensor peak torque (Nm)*								
60°/s	207.9 ± 3.4	196.1 ± 10.6	222.3 ± 4.8	219.9 ± 5.8	230.0 ± 3.7	213.4 ± 15.5	226.2 ± 6.4	208.9 ± 7.2
120°/s	166.1 ± 7.9	155.6 ± 5.9	174.3 ± 4.6	165.9 ± 5.4	179.1 ± 7.0	159.9 ± 14.1	176.7 ± 3.7	162.8 ± 5.5
180°/s	140.4 ± 9.8	134.3 ± 9.0	147.4 ± 8.0	142.7 ± 8.2	151.5 ± 15.4	123.2 ± 10.8	148.4 ± 7.1	129.9 ± 5.4
*Knee flexor–extensor strength ratio*								
60°/s	0.74 ± 0.06	0.73 ± 0.03	0.72 ± 0.06	0.66 ± 0.02	0.72 ± 0.09	0.75 ± 0.01	0.72 ± 0.05	0.71 ± 0.00
120°/s	0.75 ± 0.06	0.77 ± 0.08	0.73 ± 0.05	0.73 ± 0.04	0.73 ± 0.08	0.77 ± 0.02	0.73 ± 0.04	0.66 ± 0.02
180°/s	0.69 ± 0.01	0.67 ± 0.03	0.70 ± 0.01	0.64 ± 0.02	0.70 ± 0.01	0.68 ± 0.03	0.69 ± 0.01	0.68 ± 0.01

**Table 5:** Effects of group, velocity, and limb on maximal isokinetic strength measures of knee flexors and extensors in combat sport athletes

	(G)roup		(V)elocity		(L)imb		G × V		G × L		V × L		G × V × L	
	p	d	p	d	p	d	p	d	p	d	p	d	p	d
Maximal eccentric knee flexor peak torque	0.374	0.60	< 0.001	13.33	< 0.001	5.37	< 0.001	1.48	0.001	1.54	0.044	0.63	< 0.001	1.75
Maximal concentric knee extensor peak torque	0.003	1.39	< 0.001	20.99	< 0.001	7.29	< 0.001	2.06	< 0.001	3.46	0.411	0.31	0.013	0.99
Knee flexor–extensor strength ratio	0.010	1.20	< 0.001	1.87	0.019	0.82	0.007	1.04	0.001	1.47	0.085	0.53	0.002	1.14

**Fig. 2:**
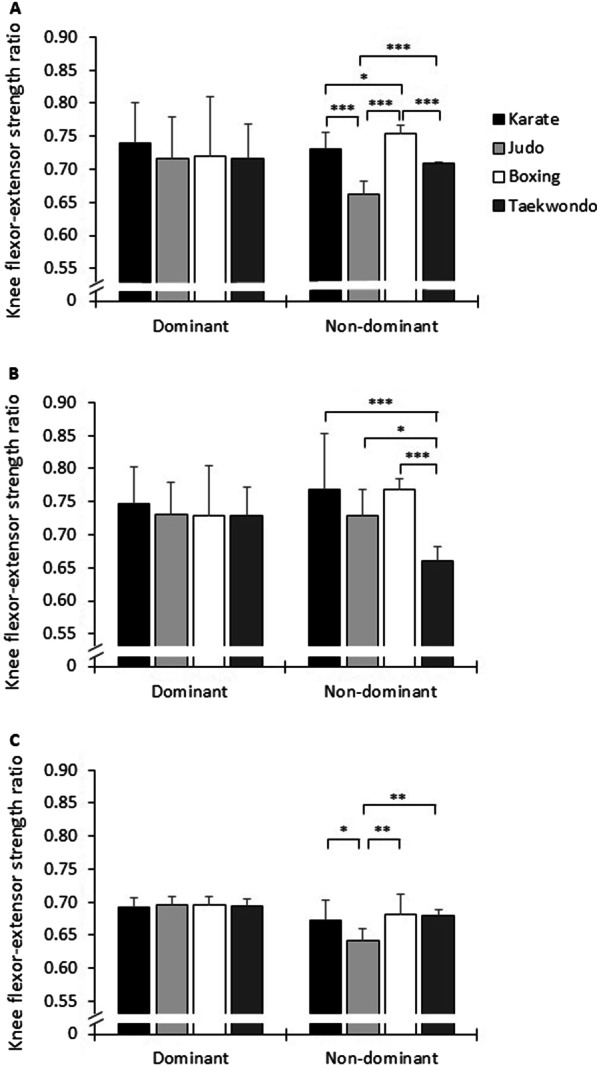
Knee flexor–extensor strength ratios at **a** 60°/s, **b** 120°/s, and **c** 180°/s on the dominant and non-dominant sides in combat sports athletes

Additionally, significant interaction effects of group, velocity, and side on eccentric KF PT, concentric KE PT, and KF–KE strength ratio were observed (*p* ≤ 0.013, 0.99 ≤ *d* ≤ 1.75). Post-hoc analysis showed larger eccentric KF PT values in amateur boxers at 60°/s (*p* ≤ 0.009, 1.28 ≤ *d* ≤ 1.77) and lower values in taekwondo athletes at 120°/s (*p* ≤ 0.034, 1.31 ≤ *d* ≤ 1.90) on the non-dominant side compared with the other groups. In terms of KE, post-hoc analysis showed lower concentric PT in karate athletes at 60 and 120°/s on the dominant side (*p* ≤ 0.028, 1.74 ≤ *d* ≤ 6.22) and lower concentric PT in amateur boxers at 180°/s on the non-dominant side when compared with the other groups (*p* ≤ 0.039, 1.12 ≤ *d* ≤ 2.03). Moreover, post-hoc analysis showed that judokas’ KF–KE strength ratios were lower at 60 and 180°/s on the non-dominant side compared with amateur boxers, taekwondo, and/or karate athletes (*p* ≤ 0.032, 1.25 ≤ *d* ≤ 5.31). Further, KF–KE strength ratios in amateur boxers were higher compared with karate athletes and/or taekwondo athletes at 60 and 120°/s on the non-dominant side (*p* ≤ 0.036, 1.16 ≤ *d* ≤ 5.87) (Fig. 2).

## Discussion

Muscle strength is an important component of physical fitness for performance success and health in many sports including combat sports [1]. Therefore, the systematic assessment of muscle strength is important to evaluate the athlete’s physical fitness level and the impact of the implemented training program. With respect to the different needs in various combat sports, upper and lower limb muscle strength measures may differ between striking (e.g., amateur boxing, karate, and taekwondo) and grappling combat sports (e.g., judo). The present study aimed to investigate maximal isokinetic upper and lower limb muscle strength measures of the dominant and non-dominant side at different angular velocities in amateur boxers, taekwondo, karate, and judo athletes. The main findings can be summarized as follows: (1) karate and taekwondo athletes revealed the lowest eccentric EF PT particularly on the non-dominant side, (2) superior concentric EE PT of amateur boxers over karate athletes shifted from non-dominant side at 60°/s to dominant side at 180°/s, (3) judoka and amateur boxers showed the largest EF–EE strength ratios particularly on the non-dominant side and at higher angular velocities, (4) eccentric KF PT was the largest in amateur boxers and concentric KE PT was the lowest in karate athletes at 60–120°/s on the non-dominant side, and (5) judokas demonstrated the lowest KF–KE strength ratios on the non-dominant side (60, 180°/s).

In terms of the upper limb muscles, the present study indicated the lowest PT measures in karate and taekwondo athletes (i.e., EF) and the largest PT measures in amateur boxers particularly on the non-dominant side (i.e., EE). These results are partly in contrast to the findings of Busko [21] and Pedzich et al. [20] who reported no differences and superior maximal isometric strength of the elbow flexors, respectively, in taekwondo athletes versus amateur boxers. The inconsistent findings between the literature and our study may be attributed to methodological reasons such as participants’ anthropometrics and/or the testing mode (i.e., isokinetic, isotonic, isometric, passive, reactive eccentric) of the device. In fact, in the study of Pedzich et al. [20] the included amateur boxers demonstrated significantly less body mass when compared with taekwondo athletes (i.e., 66 vs. 77 kg). Interestingly, peak punch force in amateur boxers was significantly and moderately associated with body mass [35]. This finding indicates that amateur boxers with higher body mass may be stronger athletes. Given that Pedzich et al. [20] reported the highest relative upper limb muscle strength measures (i.e., normalized to body mass) in amateur boxers, it appears reasonable to assume that the later have higher absolute upper limb muscle strength measures compared with taekwondo athletes of equal body mass. Additionally, using isometric testing modes as done by Busko [21] and Pedzich et al. [20] may not properly reflect the demands of dynamic muscle actions in the respective sport. However, dynamic testing such as the isokinetic modes used in the present study may better comply with the concept of training specificity [36]. Further, EF–EE strength ratios in the present study ranged between 1.17 and 1.43 indicating higher eccentric EF PT relative to concentric EE PT in combat sports athletes. Larger strength ratios were particularly observed for amateur boxers and judokas on the non-dominant side at higher angular velocities (≥ 120°/s). In general, these findings are well in accordance with isokinetic (i.e., concentric) EF/EE measurements in young-to-old male and female healthy adults [37]. Additionally, our study revealed similar EF–EE strength ratio values as earlier studies [26, 32] for the dominant and non-dominant limbs at 60 and 360°/s (concentric mode only) in male karate athletes. In contrast, Ruivo et al. [38] showed isokinetic EF–EE strength ratios of < 1.0, while Callister et al. [39] found similar isokinetic strength levels of EF and EE (i.e., EF–EE strength ratio of ~ 1.0) in male judokas. Of note, Lin et al. [31] reported an increased elbow joint injury risk for isokinetic (i.e., concentric mode only) EF–EE strength ratios of > 0.76 in male college baseball players. However, a functional EF–EE strength ratio (i.e., eccentric EF PT, concentric EE PT) was calculated in our study. It is well-known that higher PT levels can be achieved using eccentric versus concentric muscle actions [40]. This methodological difference during the isokinetic assessment protocols could explain the higher EF PT values (and the corresponding higher functional EF–EE strength ratio) observed in our study. Therefore, the athletes in our study, including amateur boxers and judokas, may not necessarily be exposed to a higher risk of elbow joint injury.

With respect to the results for the lower limb muscles, KF (*hamstrings*) and KE (*quadriceps femoris muscle*) were measured as it is considered the most multifaceted articulation in the human body [14, 41–43]. The ligaments and capsule of the knee afford stability, also it is mainly susceptible to injury especially when powers acting along the lever arms of the lower limb [44]. It has been assumed that agonist to antagonist strength imbalance can be associated with a higher risk of injury [45, 46]. Importantly, isokinetic measurements provide a truthful evaluation of muscle strength and the balance between antagonistic muscle groups (e.g., KF vs. KE) [47] and the (a)symmetry of homologous muscle groups of different limbs (e.g., KF of the dominant vs. non-dominant limb) [48]. For instance, eccentric KF strength/activity has been discussed to play an important role to decelerate knee extension movements with practical implications for performance and joint health [32, 49, 50]. In this regard, our findings of larger eccentric KF PT in amateur boxers indicated that boxers’ performance and dynamic knee stability may be better compared to the other combat sports athletes. In contrast, the lower concentric KE PT in karate athletes particularly at low-to-moderate velocities (60–120°/s) on the non-dominant side may imply that the athletes’ performance with the non-dominant leg in offensive and/or defensive techniques is less powerful. On the other hand, relying on dominant side will increase the load which might expose athletes to higher risks for injury. In addition to the single measures of eccentric KF PT and concentric KE PT, the corresponding ratio (i.e., functional KF–KE or hamstring: quadriceps ratio) appears to provide important information on the muscular balance between KF and KE [46, 51, 52] and the associated risk of overuse injury syndromes of the knee [45], ACL injuries [12, 14], and hamstring strains [53]. A balanced functional ratio (i.e., ~ 1.0) between antagonists’ and agonists’ strength levels (e.g., hamstrings vs. quadriceps) has been discussed as key factor to reduce the risk of lower limb injuries such as ACL tears [32]. In our study, the lowest functional KF–KE strength ratios were observed in judokas particularly on the non-dominant side. These ratios (0.64–0.73) were similar to the KF–KE strength ratios reported by Ghrairi et al. [54] (0.61–0.65) who examined KF–KE strength ratio (concentric mode only) of the dominant and non-dominant side (reference was the dominant arm) at different velocities in male judokas. Considering the above-mentioned relationship between functional KF–KE strength ratio and lower limb injuries, judokas may be at an increased risk for (non-dominant) lower limb injuries when compared with amateur boxers, taekwondo, and karate athletes.

Interestingly, the overall lower eccentric KF PT with higher velocity observed in our study contributes to the inconsistency in the literature [14, 32, 55–57]. In fact, while some authors reported relatively stable eccentric KF PT values across different angular velocities (30–240°/s) in athletic populations [32, 57], Evangelidis et al. [55] reported that eccentric KF PT at 50°/s was significantly larger than that at 350°/s in recreationally active individuals. Further, Deighan et al. [55] found significantly larger eccentric KF PT at 180°/s compared with 60°/s in male athletes, when tested in a seated position. However, eccentric KF PT was similar at 60 and 180°/s in a supine position. Therefore, the testing position (i.e., hip angle) appears to moderate eccentric KF strength partly explaining the inconsistent findings in the literature [14, 55].

### Limitations

This study is not without limitations. First, an inherent limitation of this study is its cross-sectional design. However, this design is considered the best way to assess multiple outcomes and determine their prevalence [58]. Additionally, cause-and-effect relations cannot accurately be drawn from cross-sectional studies. In fact, cross-sectional studies are used to inform about potential causation which should be further verified by longitudinal studies [58]. Second, the number of participants was rather low indicating that the generalizability of the findings should be considered with caution. However, an a priori power analysis was conducted and revealed that 40 participants would be sufficient to observe at least small-sized interaction effects of factors group, angular velocity, and/or limb side on maximal isokinetic KE strength (see Methods section).

## Conclusions

The present study demonstrated that maximal isokinetic elbow and knee flexor–extensor strength ratios in combat sports athletes are moderated by the type of sport, limb, and velocity. Specifically, our findings showed that amateur boxers displayed the largest concentric EE PT values on the non-dominant side potentially due to larger relative strength measures while karate and taekwondo athletes revealed the lowest eccentric EF PT values. Additionally, lower limb muscle strength was the highest in amateur boxers, whereas karate athletes showed the lowest values, particularly in the non-dominant leg, compared with the other combat sports athletes. However, in contrast to our hyporthesis, increased angular velocity did not affect group differences. Furthermore, isokinetic flexor–extensor strength ratios did not indicate different upper limb injury risks. However, in terms of lower limbs, judokas may be at an increased risk for (non-dominant) lower limb injuries due to lower functional KF–KE strength ratios compared with amateur boxers, taekwondo, and karate athletes. Knowledge of the maximal isokinetic elbow and knee flexor–extensor strength measures is of practical relevance for coaches and athletes in combat sports. It appears that competitive performance in karate and taekwondo does not require a very high level of upper and lower limb muscle strength compared with judo and amateur boxing. Moreover, it is worth to note that athletes should be trained to use both dominant and non-dominant sides to reduce the stress on the dominant side and minimize injury risk factors.

## Data Availability

The datasets generated and/or analysed during the current study are not publicly available. Upon request, the corresponding author will share the data set.

## Abbreviations

EF: Elbow flexors
EE: Elbow extensors
KF: Knee flexors
KE: Knee extensors
ACL: Anterior cruciate ligament
PT: Peak torque
TKD: Taekwondo
